# Increased self-triggered vocalizations in an epidermal growth factor-induced rat model for schizophrenia

**DOI:** 10.1038/s41598-022-17174-3

**Published:** 2022-07-28

**Authors:** Itaru Narihara, Hanako Yokoyama, Hisaaki Namba, Hidekazu Sotoyama, Hiroyoshi Inaba, Eiko Kitayama, Kota Tamada, Toru Takumi, Hiroyuki Nawa

**Affiliations:** 1 Department of Molecular Neurobiology, Brain Research Institute, Niigata University, Niigata, 951-8585 Japan; 2 Department of Biological Sciences, Graduate School of Medicine, Kyoto University, Kyoto, 606-8501 Japan; 3 Department of Physiological Sciences, School of Pharmaceutical Sciences, Wakayama Medical University, Wakayama, 640-8156 Japan; 4 RIKEN Brain Science Institute, Wako, Saitama 351-0198 Japan; 5 Department of Physiology and Cell Biology, Kobe University School of Medicine, Kobe, 650-0017 Japan

**Keywords:** Experimental models of disease, Translational research, Neuroscience, Psychology, Diseases, Medical research

## Abstract

Rats elicit two types of ultrasonic vocalizations (USVs), positive (30–80 kHz; high pitch) and negative (10–30 kHz; low pitch) voices. As patients with schizophrenia often exhibit soliloquy-like symptoms, we explored whether an animal model for schizophrenia is similarly characterized by such self-triggered vocalizations. We prepared the animal model by administering an inflammatory cytokine, epidermal growth factor (EGF), to rat neonates, which later develop behavioral and electroencephalographic deficits relevant to schizophrenia. EGF model rats and controls at young (8–10 weeks old) and mature (12–14 weeks old) adult stages were subjected to acclimation, female pairing, and vocalization sessions. In acclimation sessions, low pitch USVs at the mature adult stage were more frequent in EGF model rats than in controls. In the vocalization session, the occurrences of low pitch self-triggered USVs were higher in EGF model rats in both age groups, although this group difference was eliminated by their risperidone treatment. Unlike conventional negative USVs of rats, however, the present low pitch self-triggered USVs had short durations of 10–30 ms. These results suggest the potential that self-triggered vocalization might serve as a translatable pathological trait of schizophrenia to animal models.

Schizophrenia is one of the psychiatric disorders whose etiological and biological mechanisms are not yet known. This disorder disrupts a range of brain functions such as verbal speech and cognition, social emotion, and word construction. In particular, patients with schizophrenia often exhibit the phenotypic symptom of soliloquy or monologue during hallucination as positive symptom^1–4^. Thus, we thought the possibility that autonomous rat self-triggered vocalization might be relevant to the soliloquy-like symptom and would be useful for the translation of schizophrenia symptoms into these experimental animal models.

Various animal models of schizophrenia have been established according to genetic and/or environmental hypotheses for this disorder, and their behavioral endophenotypes have been intensively investigated^5–7^. The evaluated behavioral traits include social interaction, sensorimotor gating, working memory, and learning persistency (i.e., latent inhibition). However, there are substantial gaps between rodent behaviors and behavioral symptoms of patients with schizophrenia^8–10^. Therefore, additional behavioral endophenotypes or trait markers that match with or translate to the positive or negative symptoms in patients with schizophrenia remain to be identified^11–13^.

Based on the immune inflammatory hypothesis of schizophrenia^14–16^, we investigated animal models established by perinatal exposure to the cytokines epidermal growth factor (EGF) and neuregulin 1, which are implicated in obstetric complication or neonatal hypoxia^17–19^. Neonatal rats or mice that are challenged with EGF or neuregulin 1, which bind to ErbB receptors in the brain, later develop a variety of behavioral deficits relevant to schizophrenia^17–21^. Our latest studies revealed that these behavioral changes of this model accompany the auditory pathophysiological impairments associated with schizophrenia; reductions in mismatch negativity-like electroencephalogram (EEG) and auditory steady state response (ASSR)^22–24^. Thus, we speculated that these animal models might show the schizophrenia-associated impairments in the auditory recognition and monitoring.

Rats are known to communicate with mating partners or predators by eliciting various ultrasonic vocalizations (USVs). These voices can be grossly classified into two frequency ranges as follows: 10–30 kHz (low pitch USV) and 30–80 kHz (high pitch USV)^25,26^. Considering that schizophrenia symptoms include distorted speech and mumbling or soliloquy-like symptoms^1–4^, autonomous rat self-triggered vocalization might be useful for the translation of schizophrenia symptoms into the above EGF model. In the present study, therefore, we tested whether EGF model rats similarly produce the self-triggered vocalization. In addition, the influences of rat age, anxiety states and antipsychotic treatment on self-triggered vocalization were assessed.

## Results

### Vocalization test paradigm

**Figure 1:**
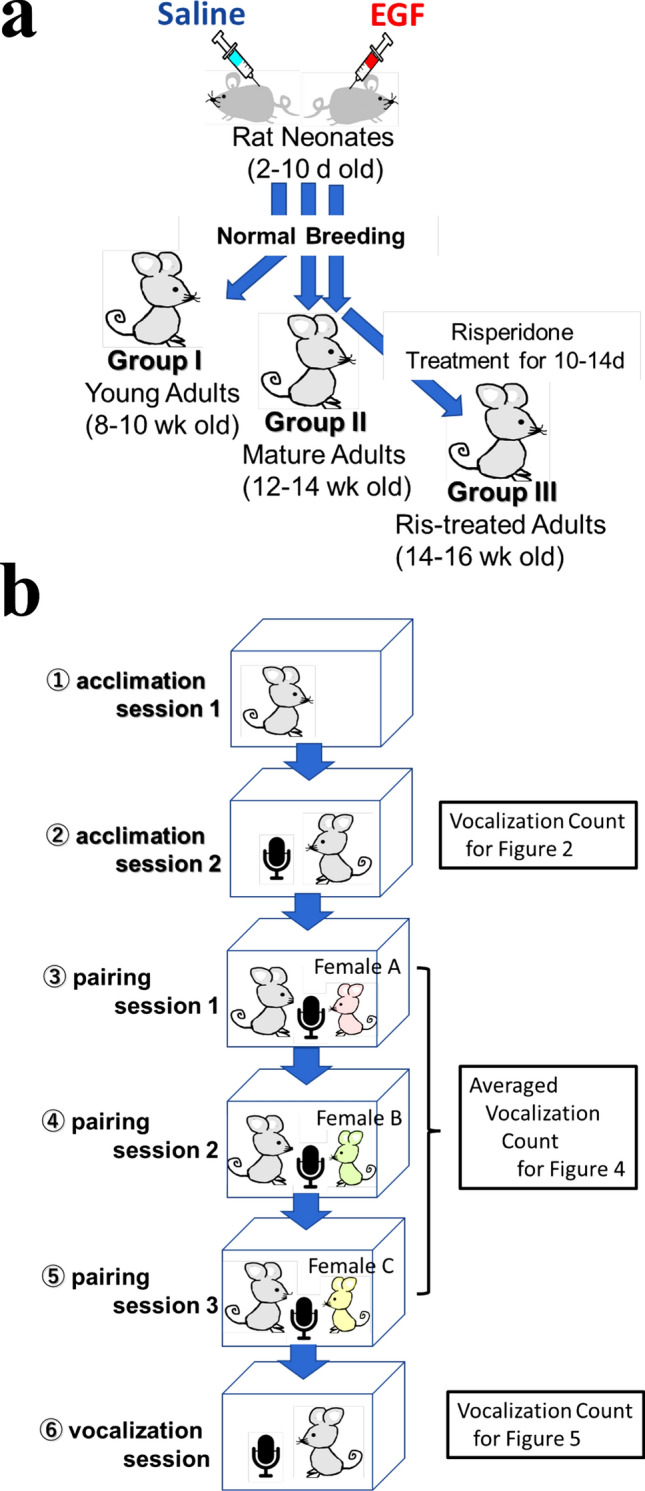
The test sequence of rat vocalization sessions. (**a**) Rat group allocation. The young (8–10 weeks old), mature (12–14 weeks old) adult rats, and risperidone-treated adult rats (14–16 weeks old) were prepared from the EGF-injected and saline-injected groups. (**b**) these rats were daily subjected to the individual test sessions. The test sequence consisted of two acclimation sessions, three pairing sessions with distinct female rats, and a vocalization session. Ris; Risperidone. Note; the same rats were not subjected to the test sequence twice.

Male rats were challenged as neonates with either EGF or saline (EGF model vs control littermates), and the EGF-treated rats served as an animal model of schizophrenia (called EGF model rats hereafter). Both groups of rats were raised until reaching young (8–10 weeks old) and mature (12–14 weeks old) adult stages. Additionally, another set of mature adult rats were prepared and subchronically treated with the antipsychotic drug risperidone (Fig. 1a). All groups of the rats were subjected to the following test sequence for vocalization measurements: (i) acclimation sessions, (ii) pairing sessions with distinct female rats^27^, and (iii) a vocalization session (Fig. 1b). As the vocalization test itself was initially anxiogenic for rats, we measured the number of self-triggered vocalizations twice at the beginning and the end of the test sequence. The self-triggered vocalizations at the end of the test sequence might be influenced by preceding male–female pairing.

### Frequent low pitch self-triggered vocalizations of mature EGF model rats during the acclimation session

**Figure 2:**
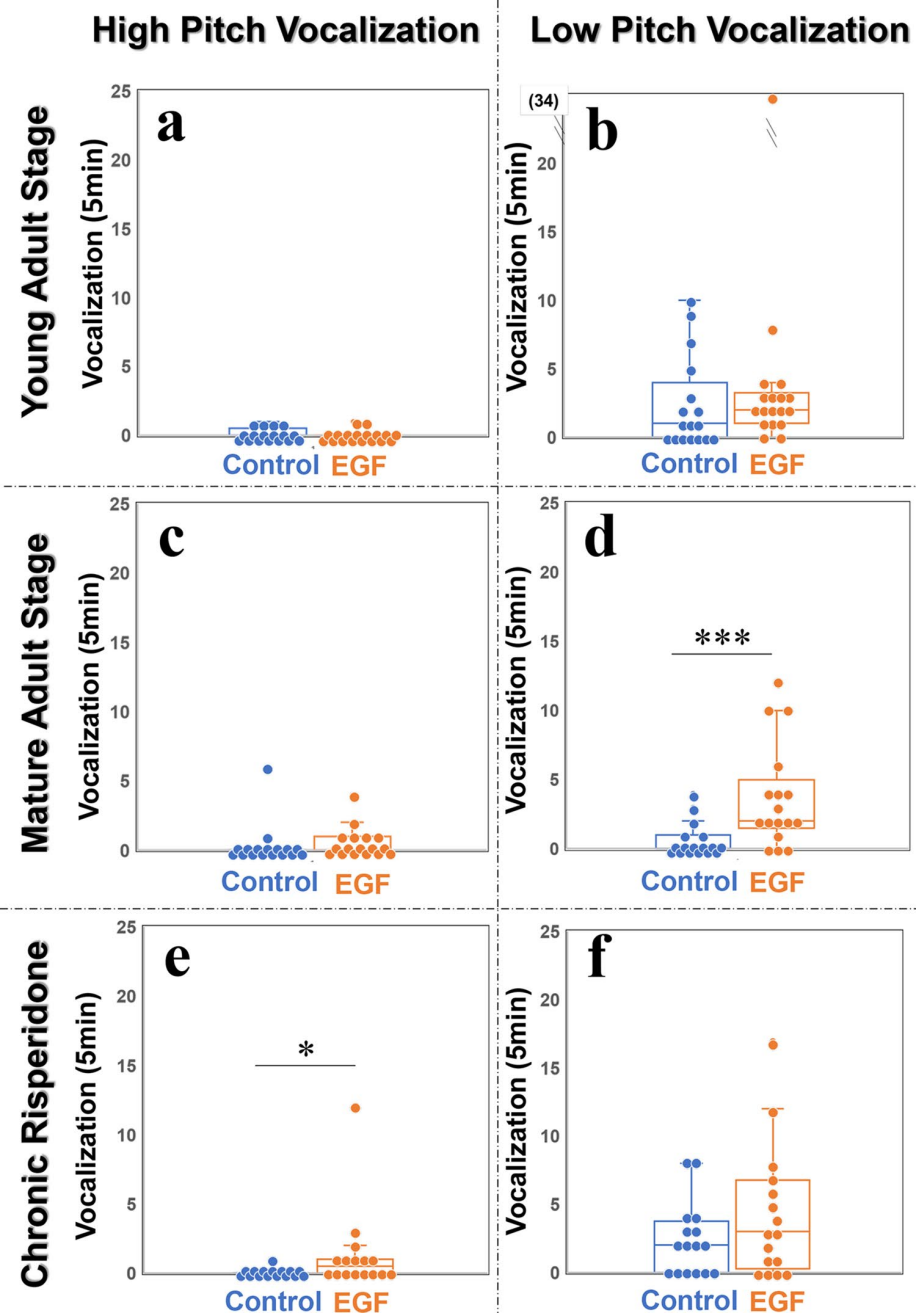
The numbers of vocalization occurrences in individual rats in the second acclimation session. The EGF model and control rats at young (**a, b**) (*n* = 17 and 18, respectively) and mature (**c, d**) (both *n* = 17) adult stages, as well as the EGF model and control rats treated with risperidone at the mature adult stage (**e, f**) (both n = 16), were acclimated twice in the soundproof recording chamber for 10 min. In the second acclimation period, rat vocalizations were recorded. The numbers of high pitch (**a, c, e**) (30–80 kHz) and low pitch (**b, d, f**) (10–30 kHz) vocalizations were counted and assigned to individual rats. One data point (34 counts) in (**b**) is located out of the present ordinate range. *q < 0.05, ***q < 0.001, by Mann–Whitney U test with Holm’s correction.

In the acclimation session, we monitored the occurrence of spontaneous self-triggered vocalizations in male rats (Fig. 2). For high pitch USVs (30–80 kHz), no significant group differences were noted between the EGF model and control rats at the young adult or mature adult stage (Fig. 2a,c), whereas the occurrence of low pitch calls (10–30 kHz) was significantly more frequent in EGF model rats than in control rats at the mature adult stage (*q* < 0.006 with Holm’s correction) (Fig. 2d), but not at the young adult stage (Fig. 2b). This group difference in low pitch calls became non-significant following the antipsychotic treatment with risperidone (*q* = 0.564 with Holm’s correction) (Fig. 2f), although the risperidone effects on control group appeared to contribute to the disappearance of this group difference. The treatment of mature adult rats with the antipsychotic drug risperidone resulted in a group difference in high pitch calls (*q* = 0.030 with Holm’s correction) (Fig. 2e).

### Distinct features of rat’s low pitch self-triggered vocalization

**Figure 3:**
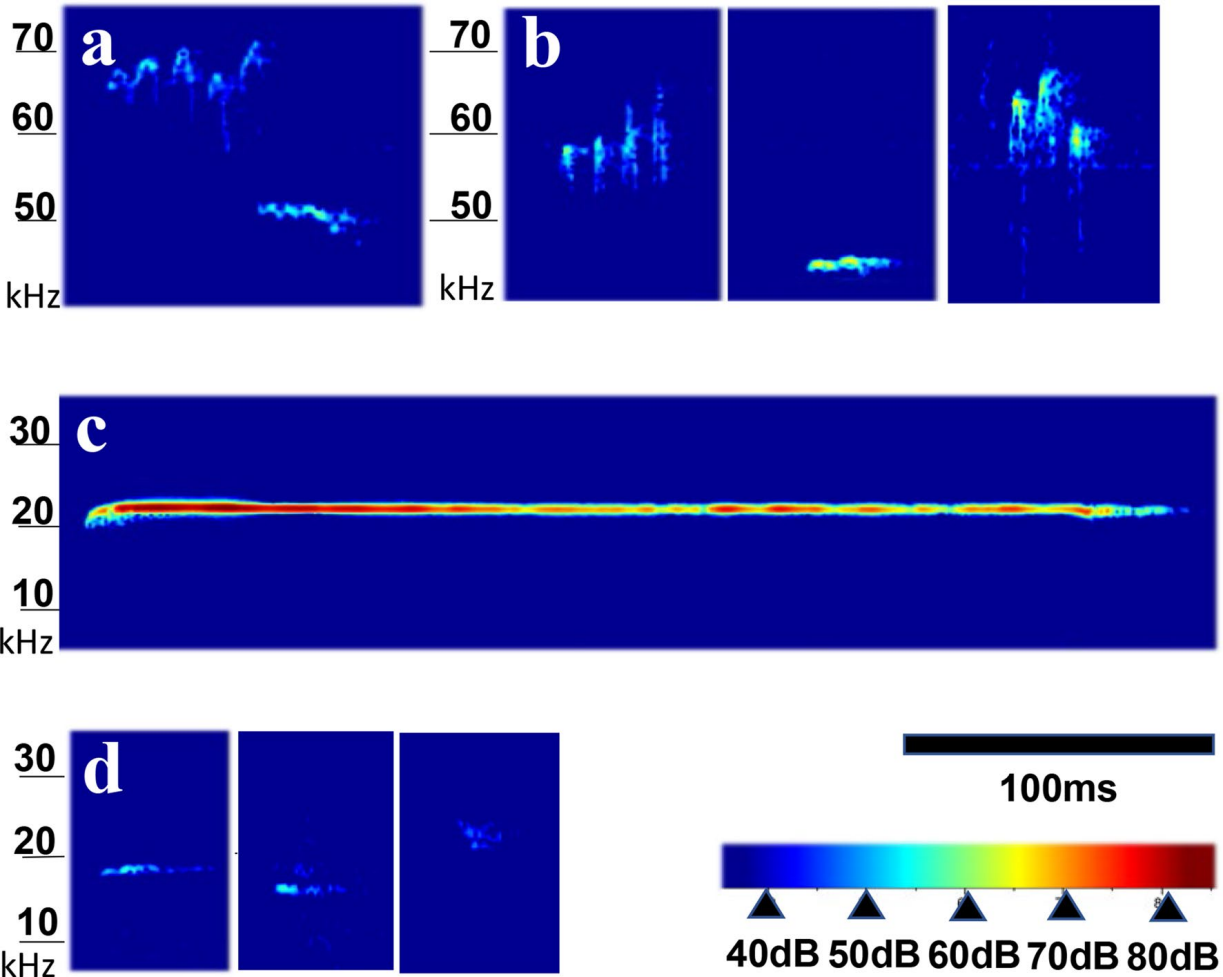
Typical time–frequency patterns of social vocalizations and self-triggered vocalizations. (**a**) A positive rat vocalization with the rapid frequency modulation, recorded in the male–female pairing session. (**b**) A high pitch vocalization, recorded during the acclimation session. (**c**) A negative rat vocalization with a flat pitch profile at 22 kHz, recorded in the male–female pairing session. (**d**) A low pitch vocalization with a short duration, recorded during the acclimation or vocalization session. For these figure display, the rat voices were analyzed with the high resolution FFT program of RAVEN PRO 1.6.1.

In addition to the quantitative difference between these groups, the self-triggered vocalizations showed similarities to and differences from the conventional vocalizations reported in rats (Fig. 3) (Table 1). The time–frequency spectrums of high pitch self-triggered vocalizations in the acclimation session had short durations with or without rapid frequency modulation (Fig. 3b). Although the mean frequency of high pitch self-triggered vocalizations in EGF model rats (40.3 ± 9.8 ms) was significantly lower than that in control rats (53.1 ± 10.3 ms) at the mature adult stage, their durations were similar (27.1 ± 11.5 ms for control and 21.5 ± 13.6 ms for EGF rats). These USV spectrums resembled those of typical rat positive voices reported previously^25,26^ and were often seen in the pairing session as well as in the vocalization session of this study (Fig. 3a).

**Table 1:** Frequency and duration profiles of rat self-triggered vocalizations.

Acclimation session	Number of events	Max frequency	Min frequency	Frequency span	Mean frequency	Mean duration
	n	kHz	kHz	kHz	kHz	ms
**High pitch calls**						
Control Rats	7	56.3 ± 10.8	50.0 ± 9.9	6.29 ± 2.98	53.1 ± 10.3	27.1 ± 11.5
EGF Rats	10	42.6 ± 9.6*	38.0 ± 10.3*	4.60 ± 3.10	40.3 ± 9.8*	21.5 ± 13.6
*Difference ( P)*		*0.019*	*0.031*	*0.28*	*0.023*	*0.37*
**Low pitch calls**						
Control Rats	11	18.3 ± 2.8	14.5 ± 3.9	3.73 ± 2.20	16.4 ± 3.2	19.5 ± 6.9
EGF Rats	62	17.1 ± 4.0	14.1 ± 4.0	3.02 ± 1.60	15.6 ± 3.9	15.3 ± 5.7
*Difference ( P)*		*0.25*	*0.74*	*0.32*	*0.48*	*0.077*

Vocalization session	Number of event	Max frequency	Min frequency	Frequency span	Mean frequency	Mean duration
	n	kHz	kHz	kHz	kHz	ms
**High pitch calls**						
Control rats	11	40.4 ± 8.2	36.8 ± 8.6	3.54 ± 1.5	38.6 ± 8.4	19.5 ± 9.9
EGF Rats	87	51.1 ± 6.5**	46.9 ± 5.6**	4.25 ± 2.7	49.0 ± 6.1**	22.7 ± 13.6
*Difference (P)*		*0.0013*	*0.003*	*0.27*	*0.0019*	*0.36*
**Low pitch calls**						
Control rats	31	19.4 ± 5.4	17.2 ± 5.7	2.16 ± 1.48	18.3 ± 5.5	16.1 ± 6.4
EGF rats	118	16.1 ± 4.5**	13.8 ± 4.5**	2.29 ± 1.36	14.9 ± 4.5**	14.6 ± 8.5
*Difference (P)*		*0.0032*	*0.0034*	*0.67*	*0.0031*	*0.28*

Self-triggered vocalizations in the acclimation session and in the vocalization session were recorded from the EGF model and control rats at mature adult stages (both 17 rats). Individual self-triggered vocalization events (n) in the rages of high pitch (30–80 kHz) and low pitch (10–30 kHz) were analyzed by the RAVEN PRO 1.6.1 program (see details in Materials and Methods). Maximum/minimum frequencies, frequency span, and duration of each event were determined and compared between groups by Welch T-test; *P < 0.05 and **P < 0.01. Raw statistical probability of each pair comparison is shown in the row of *Difference (P)*.

The typical negative voice in rats generally has long durations of more than 100 ms^28,29^ as was observed in the pairing session of the present study (Fig. 3c). Our preliminary study with the de-vocalization surgery indicated that the long-lasting low-pitch vocalization in the pairing sessions was elicited by the female rats which refused the sniffing and mounting of paired male rat (data not shown). Compared with these conventional negative voices, the low pitch self-triggered vocalization in the acclimation session exhibited markedly short durations; 19.5 ± 6.9 ms for control mature adult rats and 15.3 ± 5.7 ms for EGF mature adult rats (Table 1). The mean durations of the low-pitch self-triggered vocalizations were very short compared with that of conventional rat negative vocalizations. Our preliminary result verified the authenticity of its sound origin being the vocal cords; the low pitch self-triggered vocalization was attenuated or deformed by the local anesthesia of rat’s vocal cords with lidocaine (Supplemental Figure S1). The durations of self-triggered vocalizations in the low pitch ranges were indistinguishable between rat groups in the acclimation and vocalization sessions, but their frequency ranges were modestly different with unknown reasons (Table 1).

### Vocalization and social interaction during the male–female pairing sessions

**Figure 4:**
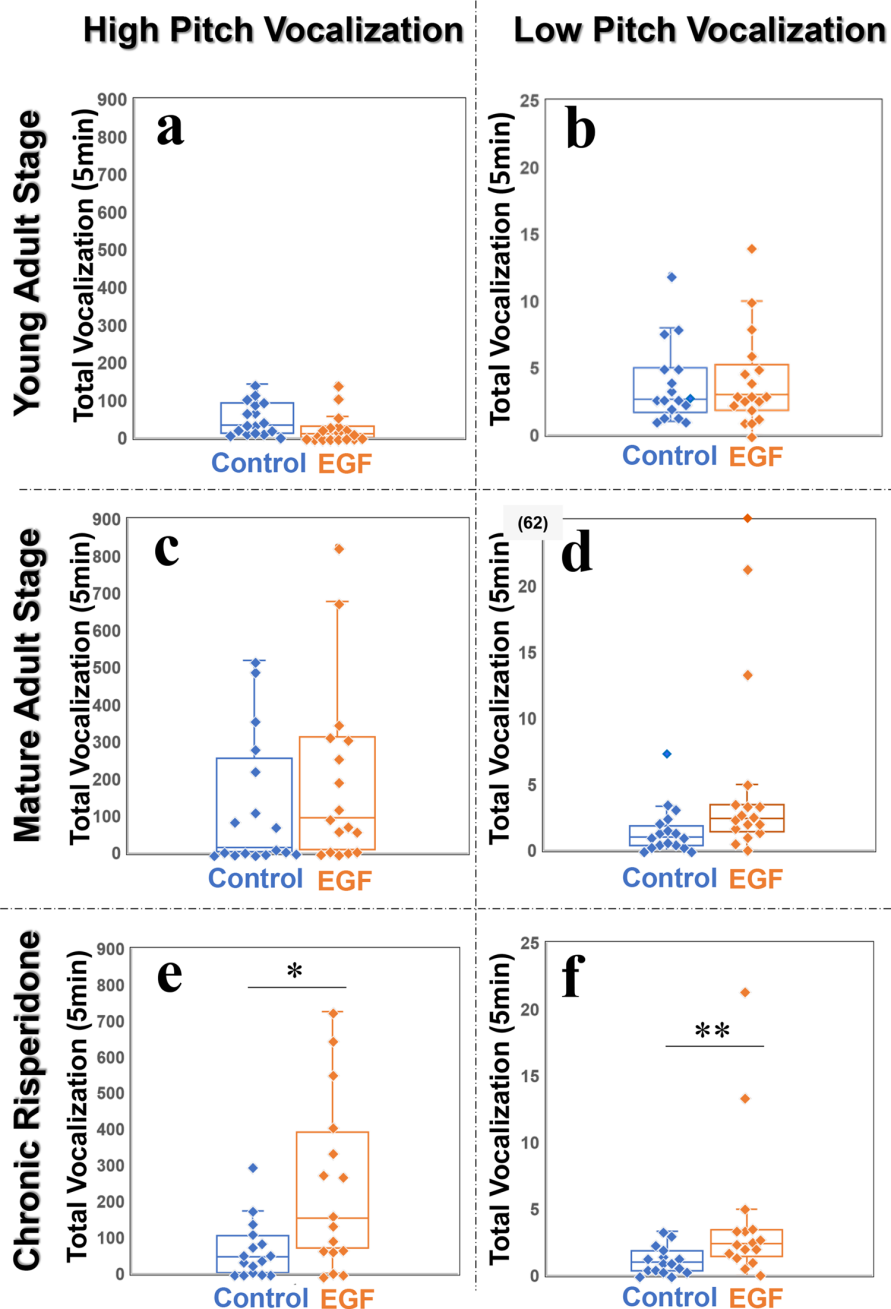
The numbers of vocalization occurrences in individual rats in the pairing session. Individual EGF model and control rats were paired one to one of individual female rats (*n* = 3). Their total vocalization occurrences were averaged over three females and assigned to each male rat. One data point (62) in Fig. 4d is located out of the present ordinate range. All other procedures are as described in Fig. 1. *q < 0.05, **q < 0.01 by Mann–Whitney U test with Holm’s correction.

In the pairing sessions, we monitored the total numbers of vocalizations elicited by both male and female rats (Fig. 4). As our aim of this study was to characterize the self-triggered vocalization of the animal model for schizophrenia, however, we did not dissect the total rat vocalization in the pairing sessions, since distinguishing the vocal origins of male and female rats was very difficult.

During the pairing session, we assessed rat social behaviors, monitoring the time male rats spent sniffing female rats. We compared these scores between the pairs of the EGF model and control rats across the three experimental conditions (young adults, mature adults, and mature adults with risperidone) using the two-way ANOVA (Table 2). The main effect of EGF was not statistically significant (F_1,77_ = 1.465; *p* = 0.230), whereas that of the experimental conditions was significant (F_2,77_ = 64.5; *p* < 0.001), although not their interaction (F_2,77_ = 1.98; *p* = 0.145). The *post*-*hoc* test revealed significant effects of the risperidone treatment; the sniffing scores of male rats were increased in the groups receiving antipsychotic treatment, irrespective of EGF administration, verifying the authenticity of the antipsychotic treatment.

**Table 2:** Durations of social interactions between male and female rats during pairing session.

	Control rats			EGF rats		
Factors	Average	SD	*n*	Average	SD	*n*
2-month *	26.4	11.2	17	25.4	13.5	18
3-month #	32.5	13.3	14	31.3	8.0	13
3-month + risperidone	62.7***^, ###^	23.0	10	76.2***^, ###^	16.1	11

The time a male rat spent sniffing a female rat was counted during the latter half of the pairing period (i.e., 5 min). The male rat was also exposed to two other female rats. A mean sniffing duration (sec) was calculated from the three pairing experiments. “*n*” represents the number of male rats examined. Two-way ANOVA with the main factors of condition (2-month, 3-month, and 3-month plus risperidone) and treatment (EGF and saline) revealed a main effect of condition (F_1,77_ = 64.5; *p* < 0.001) without an interaction (F_2,77_ = 1.980; *p* = 0.145) or an effect of treatment (F_1,77_ = 1.465; *p* = 0.230). Tukey LSD suggested *post-hoc* differences between the 3-month and risperidone groups (***p < 0.001) and between the 2-month and risperidone groups (^###^*p* < 0.001).

### Elevated low pitch self-triggered vocalizations of EGF model rats during the vocalization session

**Figure 5:**
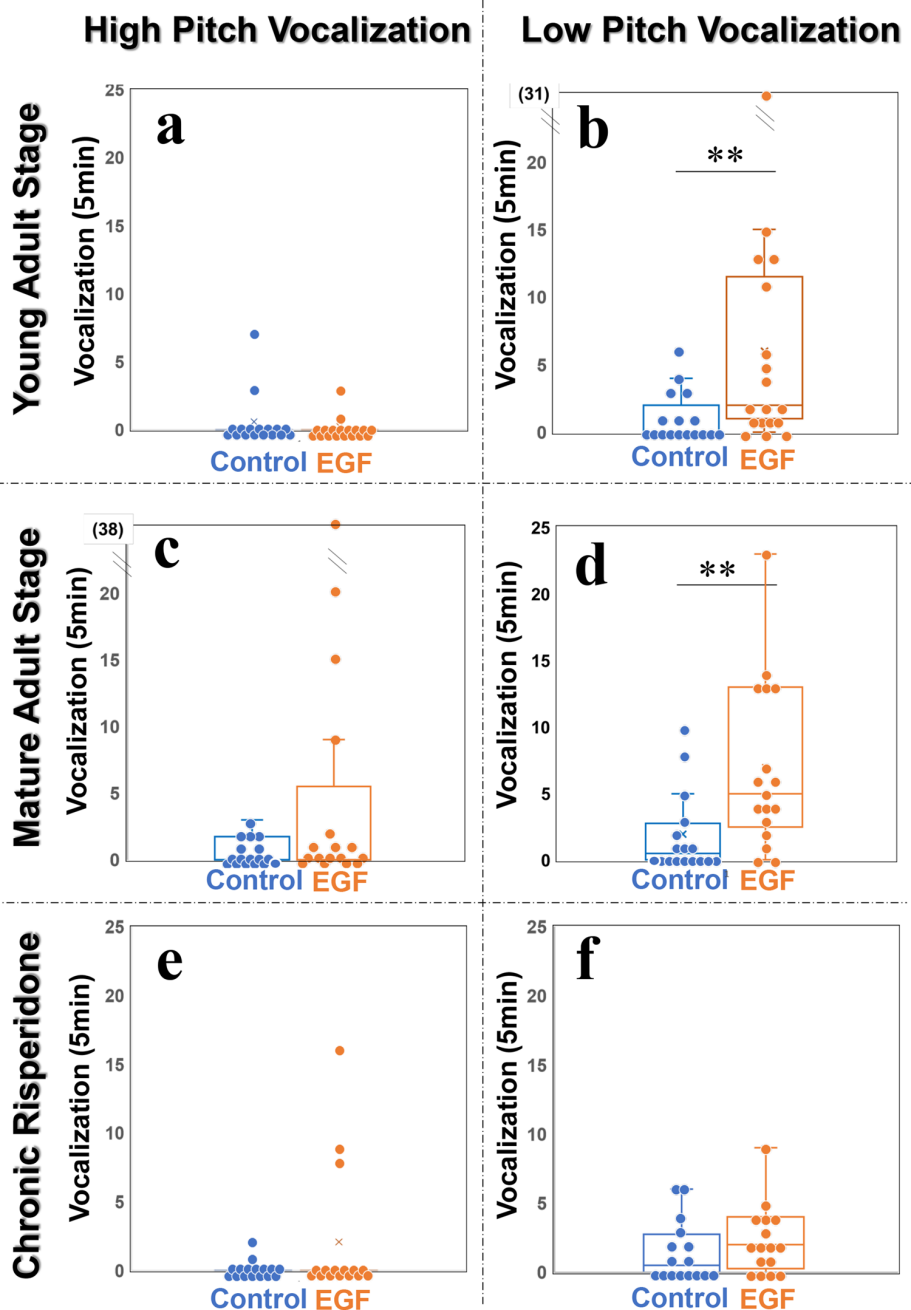
The number of vocalization occurrences in individual rats in the vocalization session. The low pitch vocalizations (10–30 kHz) and high pitch vocalizations (30–80 kHz) were counted. All other procedures and panel allocations are as described in Fig. 1, except for the number of animals tested; the number of control rats at the mature adult stage was reduced to 17. Two data points (31 and 38) in Fig. 5b and 5c are located out of the present ordinate range. **q < 0.01 by Mann–Whitney U test with Holm’s correction.

In the final vocalization session, male rats were placed alone in the same recording chamber as they had experienced in the acclimation sessions, and their self-triggered vocalizations were measured again (Fig. 5). The number of high pitch USVs was similar between the EGF model and control groups at the young and mature adult stages (*q* = 0.881 and *q* > 0.99 with Holm’s correction, respectively) (Fig. 5a,c). In contrast to high pitch USVs, the occurrence of low pitch self-triggered vocalizations exhibited significant group differences; the number of low pitch USVs in EGF model rats was significantly larger than that in control rats at the young adult stage (*q* = 0.039 with Holm’s correction) (Fig. 5b) as well as at the mature adult stage (*q* = 0.017 with Holm’s correction) (Fig. 5d). No conventional long-lasting low pitch vocalization was detected in this session. All low pitch vocalizations had short durations (Table 1). Of note, the low pitch USVs of EGF model rats appeared sensitive to the antipsychotic treatment. Subchronic administration of risperidone eliminated the above group difference of low pitch self-triggered vocalizations (*q* = 0.759 with Holm’s correction) (Fig. 5f).

To assess alterations in rat anxiety levels during the above test sequence, we monitored the number of stools dropped in each session. The numbers at the 1^st^ acclimation session were 3.58 ± 2.02 for control rats and 3.00 ± 1.55 for EGF model rats, and both values were significantly reduced to 0.08 ± 0.29 and 0.00 ± 0.00 in the last vocalization sessions, respectively (*n* = 11 or 12 each; both *p* < 0.001 between sessions, *p* = 0.773–0.902 between EGF and control groups, both by Steel–Dwass). The numbers of stool deposition indicate that rats were presumably exposed to the higher anxiety in the acclimation session but not so much in the last vocalization session.

## Discussion

In the present study, we aimed to evaluate whether the self-triggered vocalizations can be observed in the model rats in the absence of apparent external stimuli as seen in patients with schizophrenia. The major findings in the present experiment were as follows: (1) EGF model rats elicited an increased number of low pitch self-triggered vocalizations without any sensory stimulation, although the duration of these self-triggered vocalizations was shorter than the conventional negative calls^28,29^. (2) The increase in low pitch self-triggered vocalizations in the EGF group was detectable only at the pubertal stage in the high anxiogenic session (i.e., the acclimation session), but was observed at both pubertal and post-pubertal stages in the low anxiogenic session (i.e., the vocalization session). (3) Subchronic treatment with risperidone blunted the difference in low pitch self-triggered vocalizations between rat groups in the vocalization session. (4) The antipsychotic effect of risperidone was confirmed by its anxiolytic effect; the elevation of male–female social interactions. (5) The occurrence of high pitch self-triggered vocalizations was indistinguishable between the EGF model and control rats in both age groups. (6) The frequency ranges of self-triggered vocalizations differed modestly between EGF model and control groups and among sessions.

These results suggest an interesting possibility; the self-triggered vocalization of animal models for schizophrenia might serve as a translatable trait marker between human patients with and animal models for this disorder^11,30,31^, although it is fully unknown whether the self-triggered rat vocalization is relevant to the hallucination-associated symptom of the soliloquy-like mumbling. This hypothesis needs to be tested in other animal models for schizophrenia and in future studies illustrating the biological meaning of the spontaneous self-triggered vocalization of rats. Important questions remain to be addressed; whether does the rat self-vocalization reflect a communicative response or spontaneous muscle movement and whether is it elicited to self or to an imaginal partner? As some of the statistical significances were eliminated after the statistical correction for the pair-wise multiple comparisons, the present experiments will be replicated.

Low pitch USVs have a relatively long duration of 30–3000 ms with a flat pitch, referred to as negative voice^28,29^, and are generally elicited on the occasion of pain, male ejaculation, and fear; thus, they have been suggested to represent aversive emotions or alert calls^32–37^. High pitch USVs have a short duration of 5–500 ms, often with frequency modulation^38^. High pitch USVs, referred to as positive voice, are often evoked at the time of play, copulation, and hand tickling and presumably represent pleasant emotions of the rat^39–43^. The latest studies indicate that the negative and positive USVs also have communicative or social meaning to alter behaviors of neighboring rats^36,37,42^.

The duration of the low pitch self-triggered vocalizations was much shorter in its duration than that of the typical negative voice; the range of the mean self-triggered vocalization frequency was 10–24 kHz and the range of its mean duration was 6–23 ms. However, we confirmed that the occurrence of the authentic self-triggered vocalizations depended upon the rat vocal cords (i.e., their local anesthesia), indicating that the rat-evoked sounds indeed represent rat vocalizations. On agreement with our results, Barker et al. obtained a similar finding; low pitch USVs with shorter durations (10–500 ms) can be elicited by a psychostimulant, cocaine^44^. Although the biological meaning of these short low pitch vocalizations remains to be investigated further^2,35,37,41^, the latest vocalization study argues the importance of the frequency que rather than the duration que in rat emotional perception^45^.

The characteristics of the present rat self-triggered vocalizations are their stimulation-independent autonomy and their sensitivity to the antipsychotic drug risperidone. However, it is possible that the rat isolation or placement in a test chamber exerts some anxiogenic influences on rat self-triggered vocalizations. As far as we can estimate the fear/anxiety levels of rats by the number of their stools, the level was similar between rat groups and markedly decreased with the repeated sessions, becoming almost 0 at the last session. The result suggests that both groups of rats had a higher level of anxiety in the novel environment during the acclimation sessions but fully accustomed to the test environment by the last vocalization session. The fact that the number of the low pitch self-triggered vocalizations in the last vocalization session was larger than that in the acclimation session, at least, rules out the effects of anxiety from the novel test environment.

Patients with schizophrenia or amphetamine-induced psychosis also show the symptoms of the soliloquy-like muttering^1–4^. These patients unintentionally vocalize inner thoughts or speeches, often accompanied by oral motor deficits, or they have a conceptual third-person dialogue which leads to possible confusion of the self when these patients experience hallucinations^46^. This symptom appears distinct from intentional self-dialogues^3,4^. However, it is difficult to judge whether the present self-triggered vocalization of rats has any communicative functions to themselves or to other rats.

It is debatable whether the self-triggered vocalization in rodent models of schizophrenia might have neurobiological relevancy to the soliloquy-like symptoms of patients with schizophrenia. Daily systemic phencyclidine administration attenuates high pitch USVs in the absence of external sensory stimuli^47^. In contrast, amphetamine and cocaine induce behavioral traits in rodents which might be relevant to positive symptoms of schizophrenia including self-triggered vocalization^1,3^. In agreement with this, intracerebral infusion of amphetamine increases 50-kHz vocalizations^48,49^ whereas that of dopamine antagonists attenuates 50-kHz vocalizations^50^. Potasiewicz et al. reported that another neurodevelopmental model of schizophrenia presents lower numbers of 50-kHz USVs together with reduced social interaction, suggesting their relevance to negative symptoms^51,52^. In this respect, the increased self-triggered vocalizations of the present model suggest that the phenotype of EGF-treated rats is more relevant to positive symptom-like deficits of schizophrenia.

The neurobiological mechanisms underlying the low pitch self-triggered vocalizations in EGF model rats remain an open question. EGF model rats harbor hyperdopaminergic states and ectopic dopamine innervation^53,54^, which might be associated with the increase in self-triggered vocalizations. The fact that patients with amphetamine-induced psychosis exhibit persistent phenotypic symptom of muttering or soliloquy-like behaviors suggests a neurobiological association of rat self-triggered vocalizations with dopaminergic abnormalities^28,30^. However, Silkstone and Brudzynski (2020) suggest that long-lasting low pitch vocalizations of rats are resistant to dopamine agonists and antagonists but sensitive to the anxiolytic drug benzodiazepine^55^. Of note, the antipsychotic effect on low pitch vocalizations of EGF model rats was apparent in the vocalization session but not in the acclimation session. Therefore, we speculate that rat self-triggered vocalizations appear to be complex behaviors which can be influenced by various environmental conditions, emotional states, and neuropharmacological stimuli.

Although the biological and pathological relevance of high- and low pitch self-triggered vocalizations of rats to the patients’ symptom remains to be further characterized, the present observations suggest that the USV examination in rodent models is useful to explore the neurobiological fundamentals of the hallucination-associated symptoms of patients with schizophrenia.

## Materials and methods

### Animals

Neonatal male Sprague–Dawley rats (postnatal day 2) and adult female rats (7–8 weeks old) were obtained from SLC Inc. (Shizuoka, Japan). Neonatal animals (102 rats in total) with their dams and subsequently adult animals were housed in the Niigata University Animal Facility under conditions of a reversed 12-h light/dark cycle (8:00 a.m. ON and 8:00 p.m. OFF), constant temperature (23 ± 2 °C) and humidity (40–60%), with solid food and water available ad libitum. We purchased recombinant human EGF (high-purity grade; Higetsu-Shouyu, Chiba, Japan). EGF was dissolved in physiological saline and subcutaneously administered (875 ng/g body weight) into the nape of the neck of the rat pups daily from postnatal days 2–10^17^. An accelerated eyelid opening was confirmed following EGF administration, but no detrimental EGF effects on the physical development of these rats were observed. Control littermates received saline. EGF-treated rats and saline-treated controls were raised in separate polycarbonate cages (48 cm × 27 cm × 20 cm) and weaned between postnatal days 20 and 30. All behavioral examinations were performed during the day cycle. All efforts were made to minimize animal suffering and to reduce the number of animals used. However, to avoid intertrial interactions, rats experienced only one set of the vocalization paradigm. With the given use of nonparametric analyses, we needed to employ the high number of rats mentioned above.

### Vocalization recording

Male rats were subjected to each vocalization session daily between 9:00 AM and 17:00 PM. One day interval between sessions was given in their home cage, except the pairing sessions. The sessions of the male–female pairing were repeated rarely with an hour interval. Before testing, males were habituated in the test chamber for 5 min. Six vocalization sessions of 5 min each consisted of the following three paradigms: male alone (two acclimation sessions), cohousing with a female rat (three pairing sessions), and male alone (one vocalization session). In the pairing sessions, all individual males were sequentially paired with three distinct females in total to normalize the influences of the variation of female estrous cycles and male preference. As a male rat and a female rat produced USVs in pairing sessions, USVs can be attributed to either animal and thus their vocalizations were excluded from the present analyses.

### Social interaction test

In the pairing session, the behavior of the rats was video-recorded. The time duration of a male rat sniffing a female rat was determined subsequently by replaying the recorded video files. As individual male rats were paired multiple times with one of distinct female rats, the sniffing durations for each male rat were averaged prior to further statistical analyses.

### Sound analysis

Ultrasonic voices were monitored and recorded by a 1/4-inch condenser microphone (TYPE4158N, detection range 20–100 kHz; Aco Co., Ltd., Tokyo, Japan) in a recording chamber of 35 cm (D) × 40 cm (W) × 30 cm (H) dimensions (O’HARA and Co., Ltd., Tokyo, Japan). The microphone was placed 30 cm above the floor. The voice signals were transformed using a 192-kHz AD convertor (SpectraDAQ-200, Aco Co., Ltd.), processed by an on-time fast Fourier transform (FFT) analyzer (SpectraPLUS-SC; Pioneer Hill Software LLC, Poulsbo, WA, USA), and recorded in the WAV format. The sound files were further analyzed off-line using the same software with a Hanning window, an FFT size of 8192 (time resolution, 21.33 ms; frequency resolution, 23.438 Hz), an FFT overlap of 50%, an amplitude span of “–60 dB,” and a plot range of “65 dB.” The numbers of rat vocalizations were counted manually in the frequency ranges of 10–30 kHz (i.e., low pitch) and 30–80 kHz (i.e., high pitch). Alternatively, the sound files were analyzed for figure display by the RAVEN PRO 1.6.1 program (The Cornell Lab of Ornithology, Ithaca, NY, USA) with a Hanning window, an FFT size of 512 (time resolution, 2.7 ms; frequency resolution, 375 Hz), and an FFT overlap of 50% (Center for Conservation Bioacoustics, 2019).

Throat sounds elicited by respiration or sniffing as well as sound noises produced by rat movements were identified by their continuous background sound spectrums, spanning wide frequency ranges (Supplemental Figure S2) and excluded from the analysis. The frequency and number of vocalizations were scored by a trained observer who was blinded to the group allocation.

### Antipsychotic treatment

Risperidone solution (1 mg/mL; Janssen Pharmaceuticals, Tokyo, Japan) was diluted with drinking water (12.5–15.0 mg/L) and orally administered to rats for 10–14 days at a target dose of 1.0 mg/kg per day. The above-described vocalization test was performed from the 10th to the 15th day of the drug administration. Daily water consumption was monitored in each cage and was 35.1 ± 3.6 mL/day for control rats and 35.2 ± 0.6 mL/day for EGF model rats, which consequently gave rise to similar doses of 1.17 ± 0.06 mg/kg for control rats and 1.17 ± 0.02 mg/kg for EGF rats.

### Statistical analysis

Vocalization data were initially subjected to the Kolmogorov–Smirnov test and Brown-Forsy test to examine their normal Gaussian distribution and homogeneity of variances, respectively. Either or both null hypotheses were rejected in any of groups in individual sessions (data not shown). Accordingly, we applied the planned pair-wise comparisons with the nonparametric Mann–Whitney U test. With the resultant multiple pair-wise comparisons, we adjusted the *p* value of Mann–Whitney U test using the Holm’s correction method, multiplying the *p* value by the repeated number of statistical comparisons in each session and presented the Holm’s corrected *p* value as *q* value. As behavioral data from the social interaction test did not reject the above null hypothesis, alternatively, those data were subjected to two-way ANOVA with the main factors EGF/saline (two levels) and age/drug conditions (three levels), followed by the Tukey HSD as the *post-hoc* test. The number of rat stools was directly analyzed by conducting nonparametric multiple comparisons using Steel–Dwass test, and the frequency and duration profiles of rat self-triggered vocalizations were subjected to Welch T-test. Statistical analyses were performed using the SPSS software (version 11.0 J, SPSS Japan Tokyo, Japan). Data in the text represent mean ± standard deviation (SD). The alpha level for *p* and *q* values was set to be 0.05 in the present study.

### Animal ethics statement

The treatment of the experimental animals was in accordance with the local and international guidelines on the ethical use of laboratory animals. All procedures adopted in this study were approved by the Niigata University Animal Care and Use Committee and conducted under the control of the national guidelines for both the care and use of laboratory animals in Japan as well as in accordance with ARRIVE Guidelines.

## Supplementary Information

Supplementary Information.

## Data Availability

The datasets generated for this study are available on request to the corresponding author.
