# Supplementary material for: Increased self-triggered vocalizations in an epidermal growth factor-induced rat model for schizophrenia
Source: Sci Rep. 2022 Jul 28;12:12917. doi: 10.1038/s41598-022-17174-3 (PMC9334381; doi:10.1038/s41598-022-17174-3)
Supplement: Supplementary file 1 — Supplementary Information. [file 41598_2022_17174_MOESM1_ESM.docx]

***Supplemental Materials for Narihara et al;***

**“Increased self-triggered vocalization in an epidermal growth factor-induced rat model for schizophrenia”**

**Supplemental Figure S1:** Weaken or Broaden time-frequency signals of soliloquy-like vocalizations following the local anaesthetization of the vocal code.

**Supplemental Figure S2:**

Typical time-frequency map of sniffing-associated respiratory noise**s**

**Supplemental Figure S1:**

**Weaken or Broaden time-frequency signals of soliloquy-like vocalizations following the local anaesthetization of the vocal code.**

(kHz) Low-frequency range (10~40kHz)


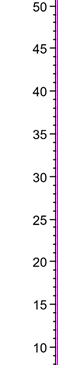

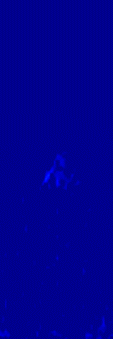

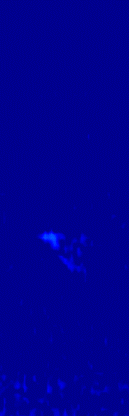

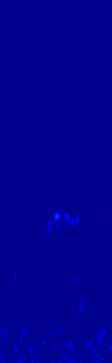

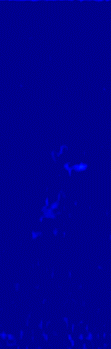

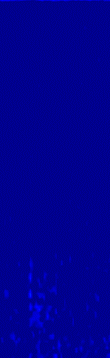


50 msec

(kHz) High frequency range (40~80 kHz)


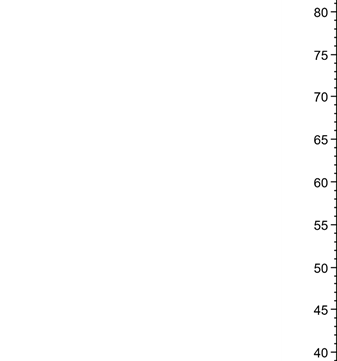

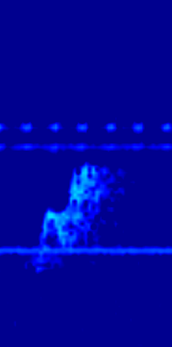

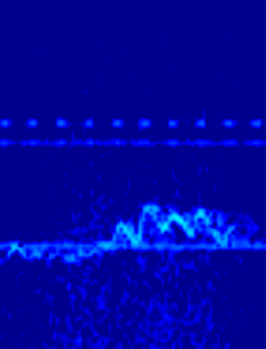


50 msec


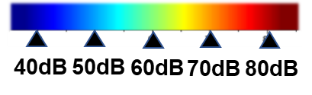


Note; the continuous background signals of 50, 62, and 65 kHz marked with arrowheads represent electric noises from a room air conditioner.

**Supplemental Figure S2:**

**Typical time-frequency map of sniffing-associated respiratory noises**

**
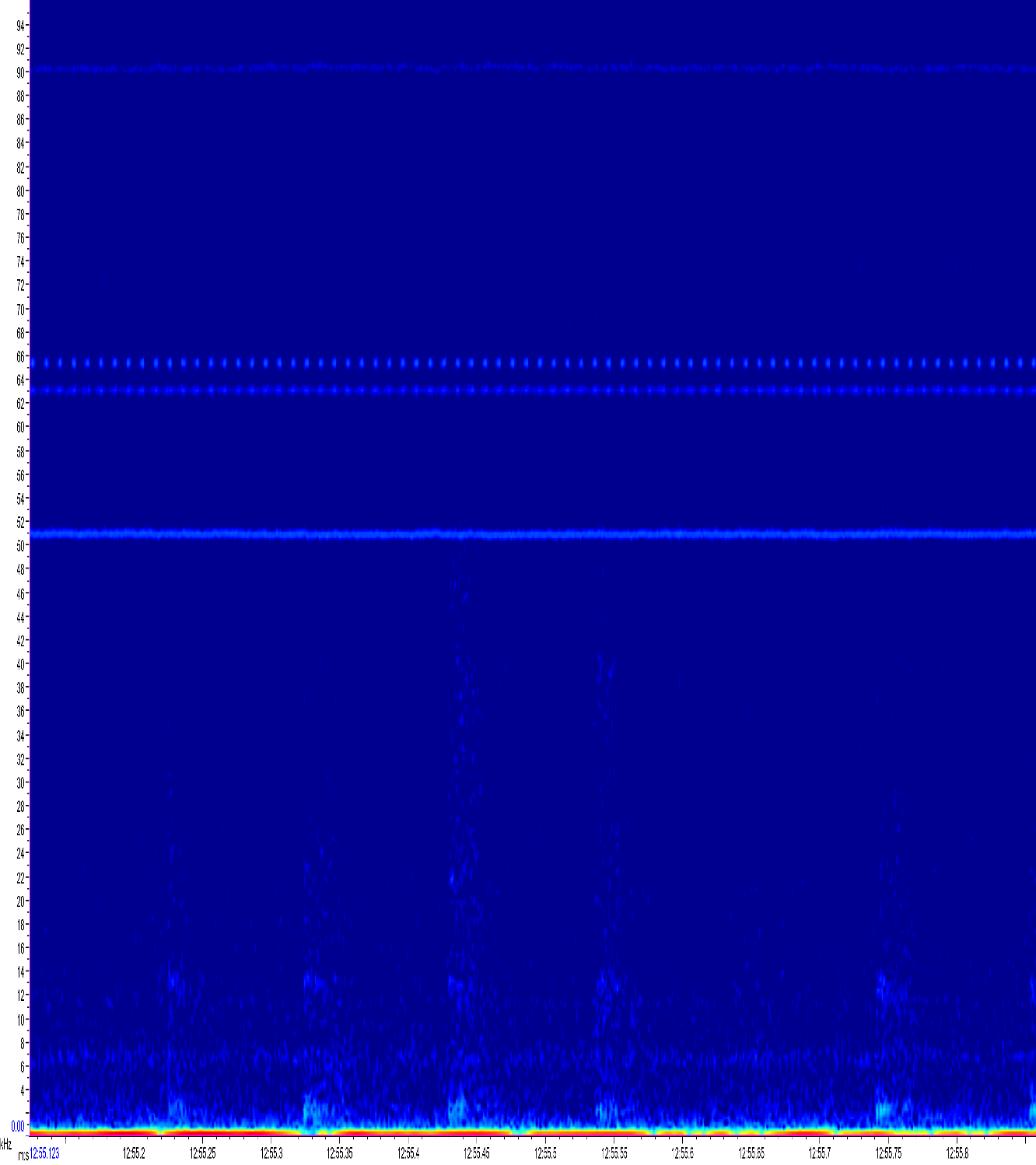
**

40kHz

20kHz

10 kHz

100 msec


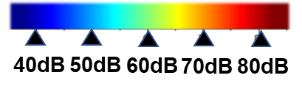


Sniffing-associated respiratory noises show the wide-range of time-frequency spectrum with a regular interval of ~100 msec.
